# Does Government Trust Matter? The Effectiveness of Policy Responses in the Health-Disaster Era

**DOI:** 10.3390/healthcare13090959

**Published:** 2025-04-22

**Authors:** Jaesun Wang

**Affiliations:** Department of Public Administration, Kangwon National University (Samcheok Campus), Samcheok-si 25913, Kangwon State, Republic of Korea; jaesunwang@kangwon.ac.kr; Tel.: +82-33-570-6631

**Keywords:** COVID-19, suppression, mitigation, government trust

## Abstract

**Background:** The COVID-19 pandemic has sparked the need for appropriate government responses in health-disaster situations worldwide. This study analyzes the impact of governments’ non-pharmaceutical measures on the number of deaths from COVID-19. In particular, it further analyzes how trust in government moderates government measures. Through this analysis, this study aims to explore the government’s appropriate role in overcoming future health disasters by understanding the effectiveness of government measures in response to the COVID-19 pandemic. **Methods:** This study collected and analyzed national data provided by various international organizations for countries of the Organization for Economic Co-operation and Development (OECD). To estimate the relationship between various policy measures and COVID-19 related deaths, it employed panel data analysis using random effects, since only three years of data, ranging from 2020 to 2022, were utilized. **Results:** The main findings of this study are as follows. First, suppression measures which include measures that place relatively stronger restrictions on people’s behavior were directly related to decreases in the number of total deaths. However, mitigation measures which consisted of weak-intensity measures were directly related to increases in the number of deaths. Second, higher levels of trust in government were directly related to decreased numbers of deaths. Finally, the moderating effect of government trust on suppression measures was not tested, but the moderating effect on mitigation measures was confirmed. **Conclusions**: This study presents the following implications. First, governments’ non-pharmaceutical measures in times of pandemic need to consider various environmental factors of the country. Second, trust in government can be an important environmental condition in overcoming health-disaster situations. In particular, trust in government weakens the side effects that can be caused by government measures. Third, suppression methods that directly affect people’s movement and daily life had a positive association with decreases in the number of total deaths, and the correlations of these measures in overcoming the health-disaster situation were confirmed.

## 1. Introduction

The COVID-19 pandemic faced worldwide since 2020 has led to an unprecedented health-disaster situation. Owing to this disaster situation, which lasted for over three years, most countries in the world have paid serious social and economic costs [1]. Additionally, COVID-19 has become an opportunity to raise public interest in the role of governments in disaster management related to the pandemic. In particular, governments of each country have tried to block the spread of infectious diseases through non-pharmaceutical interventions (NPIs). Some studies have argued that these policy interventions have had some effects, although they vary by country [2,3,4]. For example, in Germany, wearing a mask for 20 days reduced infectious disease cases by 45%, while economic costs were lower compared to other measures [5].

The main measures taken by governments have mainly focused on NPIs, aiming to alleviate the spread of infectious diseases by directly intervening in people’s daily lives and economic activities and restricting behavior. However, in contrast to their original purpose of preventing the spread of infectious diseases, these government policy responses also caused unintended consequences that severely damaged national economies and people’s income levels [6].

In 2023, the World Health Organization (WHO) officially declared the end of COVID-19 [7]. Nevertheless, the COVID-19 pandemic has sparked the need for appropriate government responses in health-disaster situations worldwide. From this point of view, research is being conducted to retrospectively evaluate the effectiveness of government policy responses in the pandemic situation [6].

This study extends these preceding studies. In other words, this study analyzes the effectiveness of the government policy responses to COVID-19, with a particular focus on NPIs.

The questions examined in this study are as follows. First, did government non-pharmaceutical responses to COVID-19 have a positive effect? Second, do the effects of various government measures differ? Finally, is the relationship between government response and infectious diseases strengthened by people’s trust in government?

This study conducts a comparative analysis between countries in the Organization for Economic Co-operation and Development (OECD). Scholars’ concern in the effectiveness of government policy has continued, but owing to differences in policy priorities and policy targets by country, conducting a comparative analysis between countries has been difficult [8]. However, the pandemic period provides an opportunity for a comparative analysis between countries on the effectiveness of the same policy which is based on the common policy priority of overcoming the pandemic worldwide [9].

Experts have pointed to the possibility of future infectious diseases even after the COVID-19 pandemic [10,11]. Understanding the effects of different governments’ various NPIs during a health disaster is expected to contribute to the search for appropriate policy responses in similar health-disaster situations in the future.

## 2. Literature Review

### 2.1. Types of Policy Responses in COVID-19 Pandemic

Government policy prescriptions for responding to the COVID-19 pandemic have been diverse. The responses can be divided into two main categories: regulation, and support. The various government responses suggested in previous studies comprise specific measures within these two categories.

Hale et al. [12] divided government actions against infectious diseases into four categories—“Containment and closure”, “Economic response”, “Health systems”, and “Miscellaneous”—with 20 specific measures presented for each category. Eight measures are included in “Containment and closure”, including “School closing”, “Workplace closing”, and “Cancel public events” “Economic response” includes economic support measures such as “Income support” and “Debt/Contact relievers for households.” “Health systems” includes seven measures, such as “Public information campaign”, “Testing policy”, and “Contact tracing.” Their classification can also be divided into “Economic response” as a support policy, and “Containment and closure” as a deterrent policy. In the case of “Health systems”, a mix of various policy types such as regulation, support, and medical measures (e.g., “Vaccination Policy”) are included.

They present several indexes by combining specific measures included in three categories. In particular, they propose the Stringency Index focusing on “Containment and closure”. Additionally, they propose the “Containment and Health Index (CHI)”, which comprises “Containment and closure” and “health systems”. Finally, the “governance response index (GRI)”, which is the broadest government response index, is also presented by adding an economic support index to CHI.

A number of studies have revealed that the government responses to COVID-19 focused more on containment policies compared to support policies [13]. The containment policies centered on social distance had the effect of weakening the spread of the initial pandemic, despite the costs they incurred, but as the pandemic continued, the socioeconomic costs caused by containment policies raised the need for governments to adjust the intensity of NPIs. Nevertheless, containment policies became an important policy tool to mitigate health disasters during the pandemic. Moreover, similar measures were adopted in most countries of the world during the pandemic’s spread [13].

Prior to the declaration of the COVID-19 virus as a pandemic by the World Health Organization [14], WHO recommended campaign-level responses, such as hand hygiene and respiratory etiquette, and restrictions on social gatherings, school, and workplace classes, as well as quantifying asymptomatic contacts, depending on the severity of the virus’ spread in each country. However, after the COVID-19 pandemic declaration, social distancing and movement control measures were recommended at the national level. These included specific measures such as prohibiting gatherings, closing schools and workplaces, and reducing public transportation.

The OECD [15] evaluated government measures conducted in major countries and included “lockdown and restrictions” as NPIs. According to the OECD, evaluations focused on measures to control the movement of people in 10 major countries, including “school restrictions”, “bans on public collections”, and “travel restrictions.”

Additionally, Rahmouni [16] presented five major ways that governments responded to the pandemic. These include movement restrictions, social distancing, state closures, public health prescriptions, and social and economic prescriptions. In particular, as government NPIs, movement restrictions, social distancing, and state closures are representative of measures used to curb virus transmission. Toshkov et al. [17] also categorized the government responses to the pandemic into three main types. School closure is a measure that forcibly closes most elementary and secondary schools. National lockdown means widespread restrictions on the movement of citizens, including prohibitions on going out, restrictions on the business of collective facilities, prohibitions on travel, and restrictions on border entry. Finally, by declaring a national emergency, the government actions institutionalized the temporary strengthening of the authority of the administration or related organizations. Excluding the declaration of a national emergency, a government’s direct interventions in the lives of its people can be divided into school and state closures.

Previous studies analyzing the effectiveness of government responses have also included similar government responses in their analyses. In this way, government measures to respond to the COVID-19 pandemic specifically suggested similar measures, such as prohibiting gatherings, restricting business, restricting movement, closing schools and workplaces, and national closures, focusing on social distancing.

Two main approaches have been used in the analyses of non-pharmaceutical measures in previous studies. The first is to measure the strength of individual measures. These studies attempted an analysis by selecting some of the non-pharmaceutical measures that governments have implemented. For example, Ji et al. [9] included school closure, workplace closure, gathering restrictions, and stay-at-home orders in their analysis model as non-pharmaceutical measures. Stanica et al. [18] also analyzed school closures, workplace closures, cancellations of public transportation, public information, and restrictions on internal movement as government policy responses to COVID-19. In addition, Mitze et al.’s [5] research analyzed the effects of face masks. All of these studies analyzed the effects of individual government measures.

The second approach used in studies is based on using existing indices. In particular, the ‘Oxford policy Strength Index (OSI)’ is commonly used [6,19,20]. OSI corresponds to ‘Continuation and closure’ among the three indicators of government response suggested by Hale et al. [12]. This includes high-intensity measures to directly limit people’s behavior. This indicator contains a total of eight sub-indicators, and OSI is the indexing of the sub-indicators. Therefore, it assesses the strength of the government measures overall rather than the strength of individual measures.

Both approaches are meaningful, but have the following limitations. The former has difficulty evaluating government measures from a holistic point of view. It is difficult to evaluate the overall effect of measures implemented by governments because not all measures are considered, with only some measures were selectively analyzed. It is also difficult to present a common direction for government responses due to the division of overly detailed measures.

In the latter approach, it is very macroscopic in that all countermeasures are indexed into one, and the overall evaluation of the government response is possible, but the difference between the measures cannot be grasped as the characteristics of the individual measures included are diverse. When a health-disaster occurs, the government has to choose different measures depending on the situation because it can be difficult to respond in a timely manner if the differentiation of the measures is not taken into account. In order to compensate for these limitations, this study intends to apply a mid-range concept that is not too microscopic or too macroscopic.

The government measures reviewed here can be divided into two conceptual categories: mitigation, and suppression strategies [21,22]. The mitigation strategies do aim not to completely block transmission but to slow the spread and promote immunity, while suppression strategies aim to reduce the number of cases by completely eliminating transmission [21]. Therefore, the suppression measures result in stronger restrictions on people than the mitigation ones. These suppression strategies are controversial in that they limit individual freedom [23]. For this reason, when the government measures based on suppression strategies are effective in responding to pandemic, this can lead to people’s compliance.

By applying the concepts proposed by Ferguson et al. [21] and González-Bustamante [22], this study divides government NPIs to cope with COVID-19 into mitigation and suppression strategies, and attempts an analysis based on these two concepts.

This distinction is intended to overcome the limitations of previous studies by classifying measures according to the difference in the extent to which the measures restrict people and by encompassing non-pharmaceutical measures that countries around the world have adopted as key measures. This study differs from previous studies in that papers applying the concepts of ‘Mitigation’ and ‘Suppression’ are very rare.

### 2.2. The Effectiveness of Government Responses

The purpose of government responses during the COVID-19 pandemic was to mitigate the spread of the pandemic and prevent infection. Additionally, the results of these government responses include decreases in the numbers of confirmed cases and deaths by COVID-19 infection. Studies analyzing the effectiveness of government responses to the pandemic have used the increase or decrease in the number of new cases or deaths caused by infection as an indicator of the measures’ effectiveness [1,6]. They conclude that the number of infected people or deaths can be regarded as effects of a government’s COVID-19 measures; however, these researchers also suggest that various indicators, such as the number of infections, deaths from COVID-19, and mortality from COVID-19, need to be considered in the assessment [20].

In addition to these outcome indicators, people’s degree of mobility has sometimes been used as the effect of government response [6,13]. Decreases in movement have been used as a measure of the degree of people’s policy compliance with suppression measures such as social distancing and an effect of the policy as an outcome variable.

Meanwhile, Dergiades et al. [1] emphasized intensity and speed in analyzing the effects of government measures. They presented the results of an empirical analysis, concluding that measures that place strong restrictions on people in the early stages are most effective in reducing the number of new cases and deaths. However, Agyapon-Ntra and McSharry [6] argued that mask-wearing is considerably more cost-effective than measures such as school closures and movement restrictions among government responses. Additionally, the influence of overseas travel controls and public information campaigns was insignificant. They analyzed the relationship between the intensity of government measures and people’s mobility. Increases in the number of infected people and deaths in the early stages of the pandemic strengthened the intensity of government intervention, and as a result, people’s mobility decreased, resulting in high compliance with government measures. However, as the pandemic continued for a long time, the degree of compliance with measures weakened and people’s mobility increased owing to public fatigue, economic damage, and vaccination. Mitze et al. [5] compared areas in Germany where mask-wearing was enforced with areas where it was not, finding that the number of infection cases tended to decrease in areas where mask-wearing was enforced.

Furthermore, a study by Zaki et al. [20] suggested that the intensity of government responses had a direct positive effect, reducing mortality caused by COVID-19. Additionally, certain measures were more effective than others [1,9]. Degiades et al. [1] argued that school closures were highly effective among the government’s various measures, while Ji et al. [9] suggested that compulsory masking was a cost-effective method.

As described above, studies analyzing the effectiveness of government responses to COVID-19 have presented various results. While some have claimed that various government measures mitigated the spread of infection, others highlighted differences by period. Moreover, some studies have pointed to differences in the effectiveness of specific measures. These differences may result from differences in the measures’ characteristics and may also vary depending on which effectiveness index is used. In other words, judging the effectiveness of government responses in health disasters implies complexity and suggests the need to carefully consider differences by response measures and period.

### 2.3. Government Trust and Pandemic Performance

Trust in the government in risk situations leads to stronger compliance with government policies. Government or political trust acts as an important factor that positively affects acceptance of government policies and reductions in risk perception [24,25,26,27,28,29].

In the COVID-19 period, which can be called a health disaster, the effectiveness of government policy responses as not due to the role of government alone, but rather, was the result of cooperation in interactions between governments and people [30]. This perspective emphasizes political trust as an important variable in explaining differences in pandemic performance between countries. The higher the political trust, the stronger the participation and compliance with government policies, leading to higher performance.

The “coproduction” perspective on explaining the relationship between trust and a policy’s effectiveness argues that the effectiveness depends not only on the government’s actions but also on the people’s acceptance and support [9]. Additionally, the relationship between the government’s actions and people’s acceptance and support exists on the basis of mutual trust. In other words, trust in the government becomes an important factor in linking the government’s actions and people’s support.

Trust in government is more important in disaster situations such as the pandemic. A review of previous studies found that trust in related organizations in a pandemic situation positively affects the public’s willingness to accept the actions recommended by the government [26]. Moreover, trust in government has an important influence on the government’s policy style and response [28]. Health-disaster situations such as the COVID-19 pandemic involve high uncertainty, and verifying the effectiveness of government measures is difficult as rapid government responses are required. In this situation, trust in the government is important to induce people to comply with the government’s policy response. Therefore, in times of disaster crisis, government trust plays a more important role than it does in normal times. In particular, trust in the government can be a key factor in the effectiveness of disaster responses as people’s lives are sensitive to and directly impacted by health disasters. Previous studies have also analyzed the relationship between the stringency and timing of government NPIs in response to the COVID-19 pandemic and the results of these responses [5,18,21,31]. Two studies focused on the causality between trust in the government and effectiveness of government response. Contrary to some studies that have investigated whether government trust affects the effectiveness of government responses, others have investigated whether the effectiveness of government response affects government trust. Some studies have verified the former’s causality but also considered the existence of inverse causality [20]. Stanica et al. [18] empirically verified that the strictness of government responses to COVID-19 affected government trust. In particular, a government’s policy responses are socially and behaviorally moderated [20,31]. These arguments imply that the effectiveness of government responses may vary depending on the policy environment.

Additionally, a positive relationship exists between compliance with government measures and trust [32,33]. Nevertheless, trust in government is not necessarily positive for compliance with government policies depending on the environment in a rapidly increasing wicked crisis situation [20].

Bollyky et al. [34] verified that the degree of trust affected the infection rate of and vaccination rate against COVID-19. They analyzed the types of trust, dividing them into ‘trust in the government’ and ‘interpersonal trust’, and they argue that both types of trust contributed to lowering the infection rate and increasing voluntary vaccination. In particular, the unique perspective of their study is that trust is divided into government trust and interpersonal trust.

Ji et al. [9] investigated whether the effects of government measures on COVID-19 were further strengthened by government trust. Their research showed that the degree of mask-wearing enforcement by the government had a positive effect on the actual rate of mask-wearing. Additionally, the degree of coercion in mask-wearing had the effect of further increasing the rate of mask-wearing by interacting with a high level of trust in government. These results imply that the level of government trust has a moderating effect on the relationship between the strength of government measures in response to COVID-19 and the actual effects. This is because government responses during a health disaster are short-term measures, but trust in government is accumulated in the long term. This means that trust in government can be an environmental condition for successful implementation of government responses.

However, in the study of Zaki et al. [20], although government trust directly affected the effectiveness of government responses, the moderating effect of government trust on government response measures and policy effects, as measured by mortality from COVID-19, could not be verified. Rather, the results were contrary to the assumption that government trust would further increase the effectiveness of government countermeasures. Zaki et al. [20] concluded that, in countries with high trust in the government, people’s autonomy is high; thus, they are more likely to feel rejected and not comply with government measures that suppress freedom. People’s low compliance with government responses is a factor that lowers a measure’s effectiveness. However, the research of Zaki et al. [20] has the limitation that it does not consider the intensity of government responses. Therefore, for their results to be more persuasive, they must be analyzed separately from government response measures. The results of the abovementioned studies show that trust in the government is an important factor in determining the effectiveness of measures.

## 3. Materials and Methods

### 3.1. Measurement

Governments have implemented various measures in response to the COVID-19 pandemic, which have been evaluated through various approaches. Many studies analyzing the effectiveness of these measures have employed the “Stringency Index” as an indicator to assess the intensity of government responses [6,13,18,20]. Similarly, Fetzer et al. [35] calculated a stringency index by incorporating measures such as school closures, workplace closures, cancellations of public events, public transportation closures, public information campaigns, and restrictions on internal movement. This approach synthesized diverse containment measures into a single composite index to assess the overall intensity and extent of government responses.

Conversely, some studies have focused exclusively on specific, relatively stringent individual measures, such as school closures, workplace closures, gathering restrictions, and stay-at-home orders [9]. Adolph et al. [36] similarly categorized government responses into measures focused on closures and isolation, including restrictions on large gatherings, school closures, restaurant closures, workplace closures, and stay-at-home orders. These studies primarily encompass responses of relatively high intensity. Additionally, some research has analyzed the effectiveness of less stringent measures, such as mandatory mask-wearing [5]. This study aims to address both stringent measures and mitigation measures.

Despite variations in scope and intensity, government responses to the COVID-19 pandemic largely fall under the category of NPIs, predominantly characterized by regulatory and containment policies. These measures have generally been assessed using two main approaches. First, many studies have adopted the “Stringency Index”, which focuses primarily on “containment and closure” measures, offering the advantage of evaluating the overall intensity of government responses. Second, some studies have measured individual policy responses, which allows analysis of the effectiveness and intensity differences among specific measures. While the “Stringency Index” provides a comprehensive view of the overall response intensity, it does not capture the unique characteristics and effects of individual measures. Conversely, focusing selectively on certain measures may overlook the diversity of government responses.

Government measures include both relatively low-intensity strategies, such as promoting mask-wearing, handwashing, and public awareness campaigns, as well as high-intensity strategies, such as social distancing, lockdowns, and mobility restrictions that impose significant limitations on daily life. Limited research has been conducted to comprehensively consider such diverse response strategies at an intermediate level of analysis. Consequently, this study adopts the concepts of suppression strategies and mitigation strategies introduced by Ferguson et al. [21] and González-Bustamante [22]. Suppression strategies aim to eliminate infections and include NPIs such as social distancing, school closures, and workplace closures, as well as restrictions on public transport, meetings, and domestic and international travel [22]. Mitigation strategies, while less intense, aim to slow the rate of infection, with policies such as isolating confirmed cases a representative example. This study intends to categorize and measure diverse government responses based on these two conceptual frameworks. By doing so, it considers both the differences in their intensity among individual responses and the overall degree of government intervention as a whole.

To measure the effectiveness of government responses, previous studies have utilized two types of metrics. The first includes indicators such as the number of COVID-19 confirmed cases, mortality, and new cases or deaths caused by the virus [1,5,6,9,20,21,22,32]. These indicators are often interpreted as the outcomes of government responses, and most studies have utilized such measures. The second type measures the degree of population mobility, which reflects the effectiveness of NPIs. Hale et al. [13] and Agyapon-Ntra and McSharry [6] evaluated how government interventions, particularly social distancing measures, limited population movement to curb the spread of the virus. While case and death indicators represent the outcomes of government responses, mobility reduction is considered a direct result. This study employs the number of total deaths and new deaths due to the COVID-19 as metrics; these have been widely used in previous studies.

Regarding the measurement of trust in government, traditional methods have involved surveys conducted at the individual level [9]. These surveys typically include questions assessing trust in national institutions or political systems (e.g., central government, local government, legislature, judiciary). For cross-national comparisons, aggregate measures at the national level are derived from individual survey data. The World Value Survey, for instance, measures confidence in government across approximately 190 countries [37]. Similarly, Zaki et al. [20] utilized Eurobarometer datasets aggregated at the national level. Following this precedent, this study uses aggregated survey data, initially measured at the individual level, to measure trust in government at the national level.

### 3.2. Data and Methods

The dependent variables of this study are total deaths per million and new deaths per million, which were derived from “Our World in Data” [38]. According to Mathieu et al. [38], the actual number of deaths from COVID-19 is likely higher than the number of confirmed deaths. This is due to limited testing, malfunctioning registration of deaths, and confusion during the pandemic. The difference between the reported new and actual deaths varies from country to country. In addition, COVID-19 deaths can be recorded in different ways from country to country (e.g., some countries can count only hospital deaths, others can include deaths at home). For this reason, the number of total deaths at a given time does not necessarily show the number of new deaths. It refers to the number of deaths reported at that time. Therefore, it is not sufficient to determine the number of deaths from COVID-19 only by the number of new deaths. The number of deaths from COVID-19 must be derived from not only in the number of new deaths but also the number of total deaths, which is the number of deaths reported, to achieve more accurate estimations. Therefore, this study, taking this into account, analyzes the total number of new and total deaths. The original data provides daily figures for total and new deaths. For cross-country comparisons on a yearly basis (ranging from 2020 to 2022), the data were converted to an annual scale. Specifically, the original data contain the counts for total deaths presented as cumulative figures on an annual basis. To obtain the total death values for each year, we utilized the cumulative total deaths count recorded on 31 December. The variable ‘total deaths’ does not represent a cumulative count across the entire three-year period. Rather, it indicates the total number of deaths recorded separately for each individual year within the three-year observation period. Subsequently, we calculated the total deaths per million by dividing the total number of COVID-19 deaths for each year by the population and multiplying the result by one million. For new deaths, we constructed the annual data by summing the daily counts of new deaths over 365 days. We also calculated new deaths per million with the same method used for the total deaths per million. In essence, both dependent variables (total deaths per million and new deaths per million) were created to represent the number of deaths or new deaths per one million population in each country.

The main independent variables are suppression and mitigation measures. We utilized data on eight different COVID-19 policy measures extracted from “Containment and Closure” indicators included in the Oxford COVID-19 Government Response Tracker (OxCGRT) [13] to construct two composite indices: suppression and mitigation measures. The eight types of government measures in response to COVID-19 utilized in this study include facial coverings (0–4 scale), restrictions on internal movement (0–2 scale), international travel controls (0–4 scale), public information campaigns (0–2 scale), cancellation of public events (0–2 scale), restrictions on gatherings (0–4 scale), stay-at-home requirements (0–3 scale), and workplace closures (0–3 scale), among others. For each indicator, a higher score on the scale represents a greater level of policy implementation. Two composite indices, suppression and mitigation measures, were constructed based on the results of factor analysis, using principal component analysis of these eight response indicators (see Appendix A Table A1). Specifically, the first component, or suppression measures encompasses six different indicators: (1) restrictions on internal movement, (2) international travel controls, (3) cancellation of public events, (4) restrictions on gatherings, (5) stay-at-home requirements, and (6) workplace closures. The other component, mitigation measures, includes facial coverings and public information campaigns. Both suppression and mitigation measures were created with factor-analytic weighted average scores, using factor loadings from the principal component factor analysis.

Additionally, as a key variable, trust in the government used data provided by the OECD and was measured as the proportion of the population aged 15 or older who trusted the government [39]. All other control variables—population over 65 years old, GDP per capita, hospital beds per thousand, and Human Development Index (HDI)—were derived from the World Bank indicators [40]. Finally, year dummy variables were also included in the analysis to control time-variant conditions over three years, while the year 2020 was set as a reference group when the deaths caused by COVID-19 were first reported.

**Figure 1:**
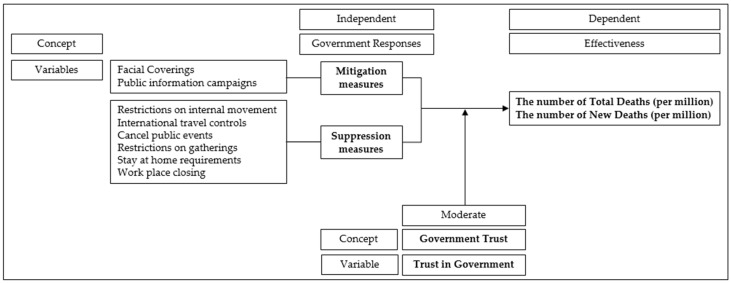
Analysis framework.

To estimate the relationship between various policy measures and COVID-19-related deaths, this study employed panel data analysis using random effects, since only three-year data, ranging from 2020 to 2022, were utilized for the analysis owing to the limited period of the COVID-19 pandemic. Additionally, the Hausman test does not reject the null hypothesis that the difference in two coefficients—fixed effects and random effects—are not systematic. However, to obtain more accurate estimates of statistical significance, robust standard errors clustered at the country level were employed in the analysis. Figure 1 presents an analysis framework for this study.

## 4. Results

### 4.1. Descriptive Analysis

The descriptive statistics summarized in Table 1 offer valuable insights into the key variables examined in this study. The total deaths reported had a mean of 1487 (SD = 1133.79), which indicates substantial variability among OECD countries. Similarly, new deaths had a mean of 746 (SD = 556.78), which also highlights differences in the distribution of newly reported fatalities.

As for the main independent variables, suppression and mitigation measures represent government responses. In this study, eight individual measures, categorized as either suppression or mitigation [21,22], were analyzed through factor analysis. Descriptive statistical analysis was conducted after the sum of scores of specific individual measures included in either suppression and mitigation were obtained. The suppression measures ranged from 0 to 18 and the mitigation measures from 0 to 6. Based on this analysis, the suppression measures had an average value of 7.23 (SD = 4.06), with a minimum value of 0.18 and maximum value of 15.04. The mitigation measures had an average of 3.84 (SD = 0.87) with a minimum value of 1.74 and a maximum value of 6.00.

These values suggest that the application of these measures varied significantly across countries. Trust in government had a mean score of 48.38 (SD = 15.75), reflecting moderate variability and providing a baseline understanding of public confidence levels in governmental institutions.

Additionally, as for control variables, economic development, measured by GDP per capita, averaged USD 38,420.32 (SD = 15,373.47). The aging population rate averaged 16.98% (SD = 4.35), while the number of hospital beds per thousand people averaged 4.47 (SD = 2.62), suggesting disparities in healthcare infrastructure availability. Finally, the Human Development Index (HDI) had a mean of 0.90 (SD = 0.05), which demonstrates a relatively high and consistent level of human development in OECD countries.

Table 2 shows the average of total deaths (per million), new deaths (per million), suppression, and mitigation measures by year. The number of total deaths was increasing over time, but the number of new deaths decreased from the highest level in 2021. In other words, 2021 was the period when the spread of the COVID-19 pandemic reached its peak. In 2022, the number of new deaths in OECD countries was 627 on average, despite the fact that the spread of the pandemic was weakening. This suggests that deaths from COVID-19 may continue to occur in the future and continued government responses are necessary.

**Figure 2:**
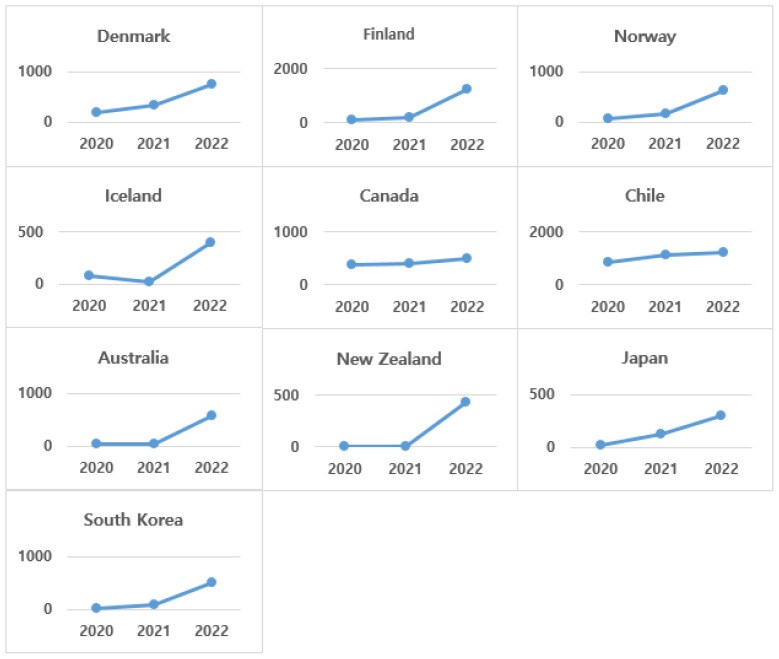
Changes in number of annual new deaths (per million)—Group 1 countries.

**Figure 3:**
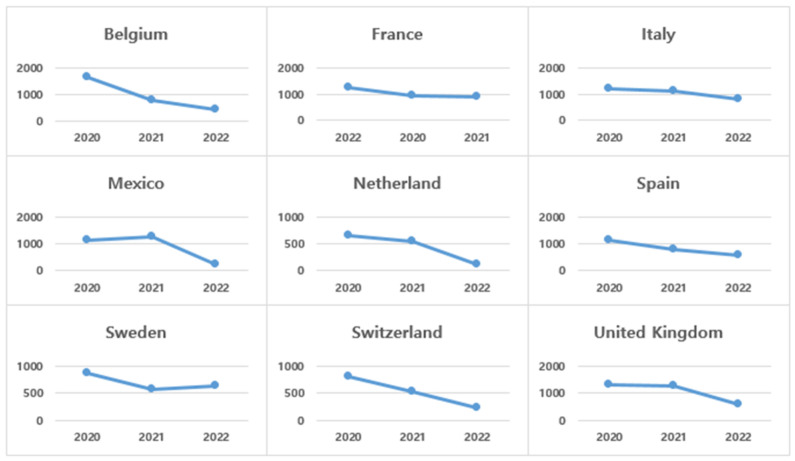
Changes in number of annual new deaths (per million)—Group 2 countries.

**Figure 4:**
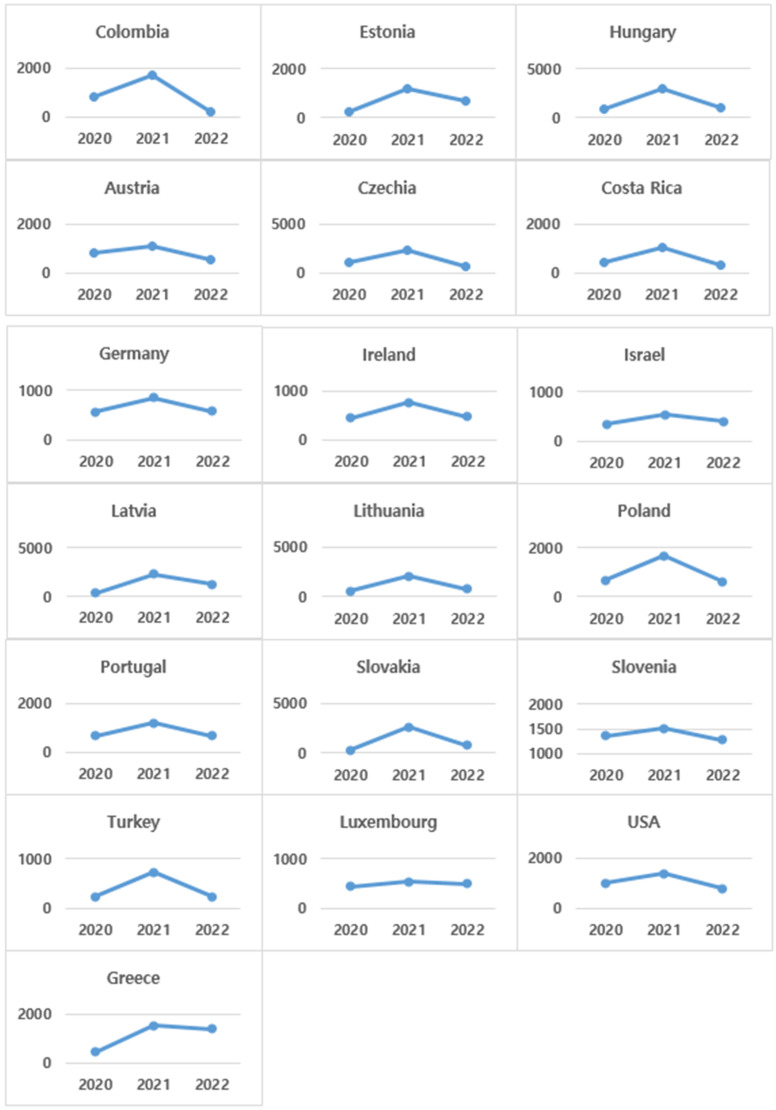
Changes in number of annual new deaths (per million)—Group 3 countries.

Figure 2, Figure 3 and Figure 4 shows the difference between countries in the distribution of the number of new deaths per million population by year (2020–2022). In terms of the change in the number of new deaths by year, OECD countries can be divided into three groups. The first (Figure 2) is the group of countries that show an increasing trend in the number of new deaths per million population. This includes European countries such as Denmark, Finland, Norway, and Iceland, and Asian and Oceanian countries. In particular, among the nine countries in this group, Nordic countries occupy the majority with four countries.

Among Asian countries, South Korea managed the pandemic with strong government-led social distancing policies in the early days of the pandemic, but the number of new deaths continued to rise from 2020 to 2022. Nevertheless, the fatality rate was reported to decrease as the years passed [41]. In addition, Iceland and New Zealand had similar numbers of new deaths in 2020 and 2021, but the numbers showed a sharp increase in 2022.

The second group of countries (Figure 3) is the countries where the number of new deaths from COVID-19 declined. Most of the countries in this group are European countries. Belgium, Italy, the Netherlands, Spain, and Switzerland had steadily decreasing numbers of new deaths over the three years, while Mexico had similar numbers of new deaths in 2020 and 2021, but the number of new deaths rapidly decreased in 2022. The UK shows a similar pattern.

The last group (Figure 4) is the countries where the number of new deaths peaked in 2021 and then decreased. Half of the OECD countries showed this pattern, making it the most common pattern in the number of new deaths by year. Omicron, a variant of COVID-19, emerged in 2021 and was recognized as the dominant virus at the end of that year. As the number of new deaths increased significantly in that year, it represents a resurgence of the COVID-19 pandemic. It can be seen that a number of east and west Euro-pean countries, as well as north and south American countries, were included in the third group.

Table 3 analyzes the average difference in the values of the dependent variables by classifying groups with below and above average values of the independent variable through a t-test. The analysis shows that the group with high trust in government had lower average total deaths and new deaths compared to the group with low trust in government, and both were statistically significant. These results show that trust in government can be an important factor in reducing the number of deaths from COVID-19. While there is a limit to directly explaining the difference in the number of deaths based on trust in government, if the public trusts the government, it is more likely to comply with policies, suggesting a possible reduction in the number of deaths.

The difference in the number of deaths between groups with above- and below-average suppression measures was found to be statistically significant. The number of total deaths (per million) was lower in the group with above-average suppression measures, while the number of new deaths (per million) was higher in the below-average group. Suppression measures that place strong constraints on people’s behavior could induce a decrease in the number of total deaths.

Finally, regarding the mitigation measures, the below-average group had lower total deaths and new deaths compared to the above-average group. In other words, for mitigation measures consisting of measures with weak constraints on people’s behavior, the effectiveness of the measures was not clearly shown. Rather, mitigation measures alone could have an adverse effect on preventing the spread of the pandemic. These results suggest that mitigation methods need to be implemented in connection with other measures rather than independently.

### 4.2. Regression Analysis

This study investigates how various policy measures are associated with the numbers of total and new deaths in a pandemic context. The regression analysis results are presented in Table 4. The impact of vaccination was not included in the regression model of this study. The number of deaths is likely to vary depending on vaccination status. However, the relationship between vaccination and the number of deaths is likely to be a reverse causality. In other words, vaccination can cause a change in the number of deaths, but an increase in the number of deaths can affect vaccination. Nevertheless, it is a limitation of this study that vaccination was not considered.

Columns 1 and 2 show that the regression estimates for suppression measures, such as lockdowns (β = −135.624 and −302.787, respectively) were negative and but statistically not significant even at *p* < 0.1. In contrast, the results for mitigation measures such as mask mandates and public information campaign, as shown in columns 1 and 2, were positive (β = 290.744 and 886.208, respectively) and were statistically significant, at least at *p* < 0.1. In addition, the estimates for trust in government (β = −13.378 and −16.248, respectively) showed a negative and significant association with the number of total deaths per million. This underscores the importance of public trust in government during health crises, likely owing to higher compliance with policies and improved communication effectiveness. Additionally, as presented in column 2, trust in government emerged as a crucial moderating variable. The results showed that countries with higher trust levels were consistently associated with reduced deaths compared to those with lower trust levels. Interaction between variables in the model provided additional insights into the dynamic between policy measures and public trust in government. Specifically, the coefficient for the interaction between suppression measures and trust in government (β = 4.389) was positive and not statistically significant at *p* < 0.1. This suggests that suppression measures are not related to public trust in government.

**Figure 5:**
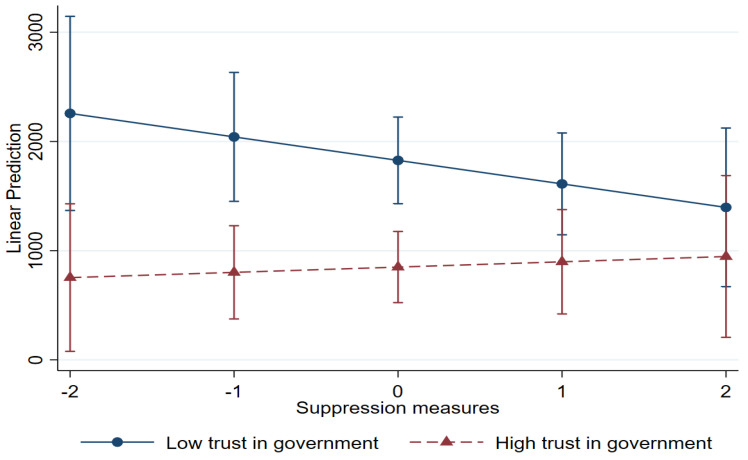
Interaction between suppression measures and trust in government: total deaths.

Figure 5 illustrates how trust in government moderates the relationship between suppression measures and the reduction in total deaths per million. When trust in government was low, it showed a slight negative trend, suggesting that suppression measures alone were related to reductions in the number of deaths. However, suppression measures were not significantly different with varying levels of trust in government, especially when suppression measures were more actively implemented.

**Figure 6:**
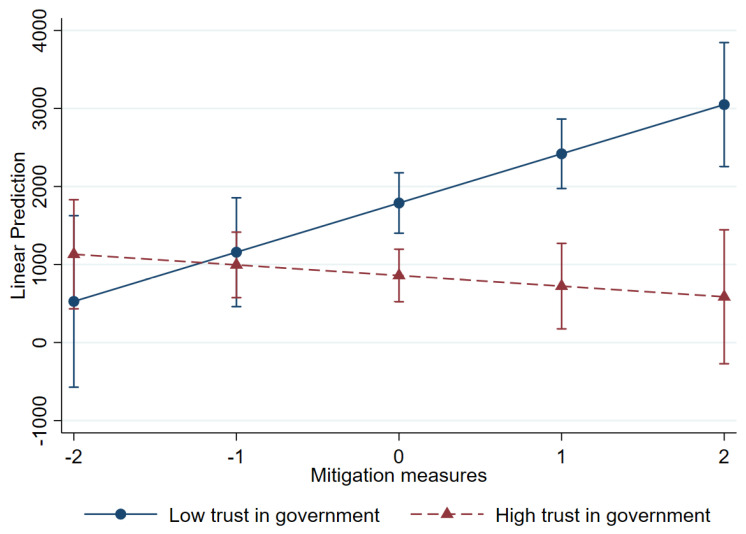
Interaction between mitigation measures and trust in government: total deaths.

Conversely, the interaction term for mitigation measures and trust in government (β = −12.781) was negative and statistically significant at *p* < 0.001, which indicates that higher trust in government weakened the positive relationship between mitigation measures and total deaths per million. Figure 6 graphically presents the interaction between mitigation measures and trust in government. It indicates that countries with high trust levels had lower mortality rates than those with low trust levels, when mitigation measures were taken. Where trust in government was especially low, mitigation measures were more related to higher mortality. This underscores the importance of public trust in government and their compliance for optimizing mitigation strategies during a health crisis.

Additionally, the coefficients for the aging population rate (β = 84.705 and 79.856) were positive and significant, at least at *p* < 0.01, reflecting the heightened vulnerability of older populations. Conversely, hospital beds per thousand and economic development showed no statistical significance, which suggests that resource availability alone was insufficient without effective governance and strategic planning. Overall, these findings emphasize the interplay of policy, public trust, and socioeconomic factors in shaping public health outcomes during crises. To sum up, trust in government significantly enhanced the success of mitigation measures, while suppression measures were not related to the reduction in total deaths, in this analysis.

Columns 3 and 4 demonstrate how policy measures are related to new deaths per million during the crisis. Specifically, suppression measures (β = −34.185) show a negative association with new deaths in column 3, but the relationship is not statistically significant. These measures were either insufficient to curb the spread of new infections in the short term or implemented reactively in areas with already rising new cases.

Additionally, mitigation measures were positively and significantly associated with new deaths. Contrary to expectations, it likely reflects the context in which these measures were introduced: regions experiencing substantial outbreaks might have adopted these measures as a reactive strategy.

Conversely, countries with higher trust in government were consistently and significantly associated with lower mortality, both in terms of total and new deaths.

In conclusion, the analysis results show that the most important variable associated with the change in the number of new deaths from COVID-19 was the degree of people’s trust in government rather than the government’s non-pharmaceutical measures. In other words, the importance of the psychological and cultural factors of trust in overcoming the health-disaster can be emphasized.

Column 4 shows the estimated interaction between policy measures—both suppression and mitigation measures—and trust in government. The estimated coefficient for the interaction term between suppression measures and trust (β = −7.652) was negative and showed statistical significance at *p* < 0.1.

**Figure 7:**
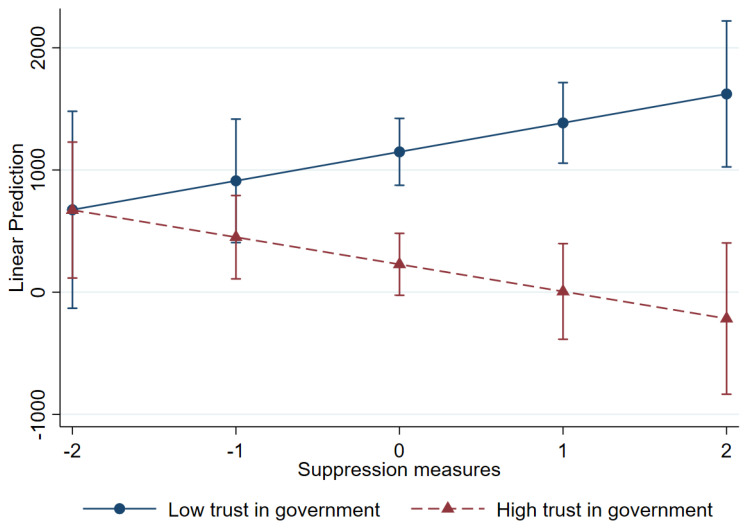
Interaction between suppression measures and trust in government: new deaths.

Figure 7 graphically shows how the relationship between suppression measures and new deaths is contingent on the level of trust in government. It indicates that where trust in government was high, suppression measures (higher than zero) were related to reductions in new mortality in the crisis. It is possible that compliance with government directives may be stronger, leading to greater adherence to restrictions and, consequently, a reduction in mortality rates. Conversely, where trust in government was low, new deaths likely increased, even with the implementation of suppression measures. Low trust in government may lead to public resistance or non-compliance, thereby undermining the intended effects of suppression policies.

**Figure 8:**
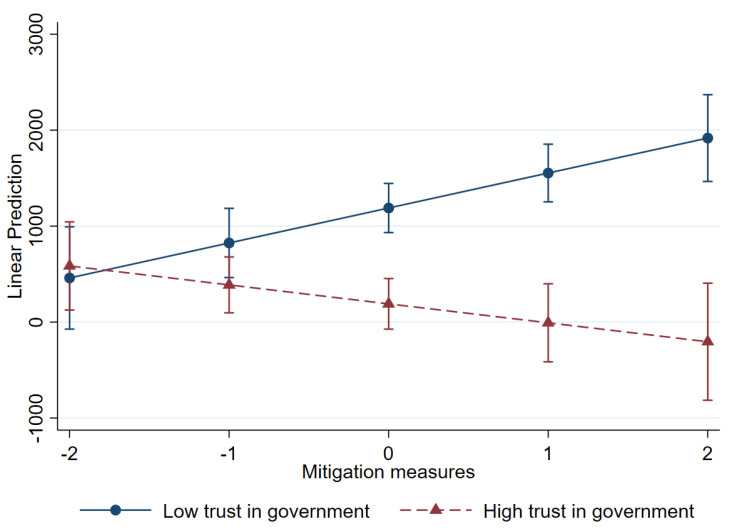
Interaction between mitigation measures and trust in government: new deaths.

The coefficient for the interaction between mitigation measures and trust in government (β = −9.364) was negative and statistically significant at *p* < 0.001. This indicates that trust in the government further strengthened the relationship between reductions in the number of new deaths and mitigation measures. When the public trusts the government, compliance with mitigation policies, such as mask-wearing and public information campaigns, is higher, decreasing the likelihood of new deaths. Figure 8 shows the relationship between mitigation measures and new deaths at varying levels of trust in government. The downward slope indicates that where trust in government was high, the effectiveness of mitigation measures improved, leading to fewer new deaths. However, where trust was low, new deaths tended to increase, even with the higher level of mitigation measures. In sum, trust in government played a crucial role in reducing new deaths when both suppression and mitigation measures were implemented. These findings reflect the critical need for governments to foster trust to maximize the impact of policies during health crises.

Considering the control variables, a higher aging population rate was strongly associated with increased new deaths, and HDI was negatively related to the number of new deaths per million. However, hospital beds and economic development were not statistically significant in the models. Overall, mitigation measures were likely to be reliant on trust in government in controlling new deaths, which emphasizes the need for building public trust in a crisis.

## 5. Discussion

This study aims to theoretically verify the effectiveness of government policies. Policies are defined as “actions that the government decides to do or not do” [42], “government activities on the premise of social change to solve problems” [43], and “Future Action Guidelines determined by government agencies” [44]. Fundamentally, policies are the government’s active or passive actions for problem solving.

Presenting generalized results based on a comparison of national policies was difficult owing to the specificity of national situations and diversity of policy problems and measures. However, the COVID-19 pandemic situation was a common health-disaster situation experienced worldwide, and the response of each country was also characterized by similar prescriptions, albeit to different degrees. Therefore, presenting generalized results on the effectiveness of government non-pharmaceutical measures to cope with the pandemic may be reasonable.

Therefore, this study analyzed how governments’ non-pharmaceutical measures were related to the number of deaths from COVID-19 and verified the moderating effect of government trust as a situational variable. As a result of the analysis, several topics for discussion arise.

First, this study categorized government responses to COVID-19 policies into suppression and mitigation measures, revealing differing effects for each type. Specifically, suppression measures had no significant relationship with reductions new deaths or reduction in total deaths. In contrast, mitigation measures exhibited consistent and significant relationships with changes in both total deaths and new deaths. These findings suggest that government policies, such as mandatory mask-wearing during the early stages of the pandemic, might not have been highly effective. Instead, they imply that the failure to implement appropriately timed and adequately scaled policies could be associated with increases in mortality.

Second, trust in the government is negatively associated with the numbers of total deaths and new deaths. Nevertheless, simply interpreting that trust in government has a direct relationship with the number of deaths has some limitations [20]. The outcome of reduced numbers of deaths in countries with high trust in the government may be due to high compliance with government measures. The findings of Ji et al. [9] support these results, confirming that trust in government increases compliance with government measures. Additionally, the effect of trust in government must be explained based on each country’s various specificities [20]. Therefore, discussing the various characteristics of each country along with trust in government is necessary.

Third, the issue of distinguishing between types of trust should be considered. Bollyky et al. [34] classifies trust into trust in government and trust in people, and reports that both types of trust affect reductions in the infection rate. The concept of trust is multidimensional and dependent on the object. These results suggest that depending on the object of trust, it may have a differential effect on mortality or infection rates. Although this study focused on trust in government, future studies need to consider the concept of multidimensional trust.

Finally, this study finds that trust in government has a statistically significant moderating effect on new deaths. Suppression measures alone were not significant for reducing new deaths; however, the relationship between suppression measures and new deaths was contingent on public trust in government. In addition, the moderating effect of government trust on mitigation measures is statistically significant with regard to both total and new deaths, showing a negative effect. Mitigation measures show a direct effect on the increase in the number of total and new deaths, but where trust in government is high, the mitigation measures are more likely to be effective in reducing the number of deaths. Strong lockdowns implemented by governments have no moderating effect on trust, but weak lockdowns have a moderating effect [20]. Additionally, the interaction effect of government trust in compulsory mask-wearing measures has been verified [9]. According to the results of this study and previous studies, government trust, in combination with weak-intensity measures, suppressed increases in the number of deaths. Since weak-intensity measures were mainly implemented at the beginning of the pandemic, the effect of government trust needs to be considered with regard to the stage of the spread of the pandemic.

## 6. Conclusions

This study aimed to investigate the effectiveness of non-pharmaceutical measures implemented by the government during the COVID-19 pandemic by conducting an analysis using panel data for three years for OECD countries. The analysis results have several implications. First, governments around the world implemented similar measures, such as social distancing, to respond to the spread of the pandemic. Nevertheless, the range and intensity of responses varied by country. In other words, the response to the pandemic varied according to each country’s specific characteristics. These results suggest that non-pharmaceutical measures of countries in pandemic times need to consider the country’s various environmental factors.

Second, trust in the government had a positive effect on the decrease in the number of deaths and also a moderating effect on mitigation measures. The results show that trust in government can be an important factor in overcoming health-disasters such as COVID-19. Mitigation measures had a positive effect on the increase in the number of total and new deaths. These results conflict with the expectation that both measures will reduce the number of deaths; rather, they may have the side effects of increasing the number of deaths.

In particular, mitigation measures, which consist of mask-wearing and campaigns, independently increased the number of deaths. Mitigation measures were recommended or enforced by the governments of each country in the pandemic’s early stages. Since mask-wearing causes inconvenience to people, resistance to its enforcement appeared in some regions [9]. These people’s discomfort and resistance weakened their compliance with mitigation measures in the period before the pandemic spread in earnest. This phenomenon might have resulted in increases in the number of deaths by failing to prevent the spread of the pandemic.

However, in countries with high trust in the government, people’s compliance with the government’s mitigation measures can increase. Additionally, high compliance with government policies can weaken the increase in the number of deaths. Therefore, the importance of accumulated trust in the government among people as well as the government’s direct measures to cope with the pandemic were confirmed. Moreover, the large effect of trust in the government can vary depending on the intensity of non-pharmaceutical measures and the stage of the spread of the pandemic. Weak-intensity measures such as wearing a mask are likely to be implemented in the early stages of the pandemic, and the higher the trust in government, the higher the compliance with weak-intensity measures. As a result, such measures can reduce the increase in the number of deaths caused by the pandemic.

The above results suggest that the overall effectiveness of OECD countries’ measures in response to the COVID-19 pandemic was limited, but trust in government was directly or indirectly related to the number of deaths. Therefore, how the government gains people’s trust can act as an important environmental condition in overcoming the crisis in a time of health disaster. Additionally, the effectiveness of each measure may be differentiated by the period of the pandemic, and the mechanism of effectiveness may vary. Trust in government was relevant in reducing the side effects of measures that weakly constrained people in the early days of the pandemic. But at the height of the pandemic, measures that strongly constrain people’s behavior can become more directly relevant. The government needs to establish a differentiated response strategy according to the timing and spread of the pandemic, and the degree of public trust in the government.

This study has several limitations. First, the classification of the timing in the analysis was excessively simple. Responses to COVID-19 can involve short-term variations on a monthly or daily basis as well as on an annual basis. Therefore, changes in government response could be more accurately grasped by further subdividing and analyzing the period of the pandemic. This study had the limitation that more detailed analysis of the government’s response during the COVID-19 period was impossible because, except for the government response variables, only annual data were available for other variables. 

Next, vaccination was not considered. The situation before and after vaccination may show differences with regard to the effectiveness of non-pharmaceutical measures, but our analysis did not reflect this. Finally, inverse causality must be considered. This study assumed a causal relationship between the strictness of the government measures and the number of deaths, but some studies have found an inverse causal relationship between the number of deaths and government measures [9]. In other words, as the number of deaths increases, the strictness of the government’s NPIs becomes stronger. Additionally, one study found that government trust increases when the number of deaths from COVID-19 decreases [20]. Future studies must investigate the inverse causality between these government measures and the number of deaths.

## Figures and Tables

**Table 1:** Descriptive statistics.

Variables	Min.	Max.	Mean	S.D.
Total deaths (per million)	4.82	4859.79	1487.02	1133.79
New deaths (per million)	4.82	2935.60	746.43	556.78
Suppression measures	0.18	15.04	7.23	4.06
Mitigation measures	1.74	6.00	3.84	0.87
Trust in government	17.15	84.63	48.38	15.75
Economic development	13,254.95	94,277.96	38,420.32	15,373.47
Aging population rate	6.86	27.05	16.98	4.35
Hospital beds (per thousand)	1.13	13.05	4.47	2.62
Human Development Index	0.77	0.96	0.90	0.05

**Table 2:** A three-year average comparison.

Variables	2020	2021	2022
Total deaths (per million)	609.64	1612.35	2239.07
New deaths (per million)	609.64	1002.71	626.94
Suppression measures	9.47	10.10	2.13
Mitigation measures	3.33	4.56	3.63

**Table 3:** t-test results.

Independent Variables		Dependent Variables			
		Total Deaths (per million)	t-Value	New Deaths (per million)	t-Value
Trust in Gov.	below avg.	1702.00	3.54 ***	983.13	4.64 ***
	above avg.	980.18		508.27	
Suppression	below avg.	2056.12	4.08 ***	594.17	−2.61 **
	above avg.	1155.05		835.25	
Mitigation	below avg.	1015.34	−4.12 ***	528.22	−4.12 ***
	above avg.	1842.59		910.93	

Note: *** *p* < 0.001, ** *p* < 0.01, * *p* < 0.05.

**Table 4:** Panel data regression analysis results.

	Total Deaths (per million)		New Deaths (per million)	
	(1)	(2)	(3)	(4)
Suppression measures	−135.624	−302.787	−34.185	390.056
	(133.504)	(241.802)	(96.610)	(238.630)
Mitigation measures	290.744	886.208 **	102.510	551.695 ***
	(164.769)	(276.184)	(84.105)	(149.403)
Trust in government	−13.378 *	−16.248 **	−16.257 ***	−15.320 ***
	(6.243)	(4.946)	(4.077)	(4.028)
Suppression measures × Trust in government		4.389		−7.652 ^†^
		(3.842)		(4.022)
Mitigation measures × Trust in government		−12.781 ***		−9.364 ***
		(3.564)		(2.696)
Economic development	820.277	749.117	582.502	593.782
	(908.938)	(871.221)	(404.544)	(423.655)
Aging population rate	84.705 **	79.856 **	55.843 ***	49.649 **
	(26.981)	(26.697)	(14.138)	(16.496)
Hospital beds (per thousand)	−54.163	−45.348	−50.298	−29.980
	(51.287)	(52.460)	(28.127)	(32.670)
Human Development Index	−12,654.656	−11,960.880	−6587.950	−6454.345
	(7487.267)	(7331.710)	(3683.646)	(3827.781)
Year 2021	443.738	511.743	173.194	196.801
	(355.856)	(345.869)	(213.076)	(195.741)
Year 2022	994.931 *	1159.508 **	−149.972	−15.045
	(390.674)	(403.362)	(198.804)	(185.599)
Constant	3262.998	3454.582	653.227	276.521
	(3810.904)	(3512.675)	(1413.619)	(1541.312)
R^2^	0.57	0.61	0.38	0.45
Number of observations	98	98	98	98

Note: *** *p* < 0.001, ** *p* < 0.01, * *p* < 0.05, ^†^ *p* < 0.1.

## Data Availability

The datasets supporting analysis of this article are included within the article and references.

## Appendix A

**Table A1:** Factor analysis.

Component	Suppression	Mitigation
Facial coverings	0.409	**0.748**
Restrictions on internal movement	**0.787**	−0.002
International travel controls	**0.807**	−0.056
Public information campaigns	−0.239	**0.858**
Cancellation of public events	**0.945**	0.026
Restrictions on gatherings	**0.876**	0.068
Stay-at-home requirements	**0.873**	0.055
Workplace closures	**0.933**	0.139

Notes: extraction method: principal component analysis; rotation method: Varimax with Kaiser normalization; rotation converged in three iterations.
